# Level of health laboratory service quality, service interruptions, and its predictors in public hospitals in Harar town, eastern Ethiopia

**DOI:** 10.3389/frhs.2024.1492766

**Published:** 2024-11-19

**Authors:** Dire Zakir, Getachaw Kabew Mekonnen, Belay Negash, Dadi Marami

**Affiliations:** ^1^ School of Medical Laboratory Science, College of Health and Medical Sciences, Haramaya University, Harar, Ethiopia; ^2^ School of Public Health, College of Health and Medical Sciences, Haramaya University, Harar, Ethiopia

**Keywords:** eastern Ethiopia, interruption, laboratory service, predictors, quality

## Abstract

**Background:**

Quality laboratory services are key in the healthcare system for successful diagnosis and patient care. Uninterrupted laboratory services are needed to meet the needs of all patients and clinical personnel, but studies in developing nations revealed that most clinicians were dissatisfied due to the lack of quality laboratory services and frequent interruptions.

**Objective:**

This study aimed to assess the level of health laboratory service quality, service interruptions, and its predictors in public Hospitals in Harar town, eastern Ethiopia.

**Method:**

A facility-based cross-sectional study was conducted at Hiwot Fana Specialized University Hospital and Jugel General Hospital between January and April 2024. Data collection utilized Standardized Stepwise Laboratory Improvement Process Towards Accreditation (SLIPTA) checklists and questionnaires based on the Ethiopian Hospital Standard Transformation Guidelines. Data were entered and analyzed by Statistical Package for the Social Sciences, version 26. Descriptive statistics such as frequencies, proportions, and means, were calculated. Binary and multivariable logistic regression models were applied to identify predictors with adjusted odds ratio (AOR) and a cut-off *p*-values <0.05 with 95% confidence interval (CI).

**Results:**

Two public hospital laboratories and their 54 laboratory professionals were involved in our study. According to our study, the mean score of the two public hospitals was 168.5 (61%), while Hiwot Fana specialized university hospital scored 212 (77%) and Jugel General Hospital scored 127 (46.1%). The study also revealed that out of 72 tests, 31 (43.05%) were interrupted, with clinical chemistry tests being the most interrupted (16, 51.6%) followed by Serology (7, 22.6%) and Hematology (4, 12.9%) tests. Lack of timely management response (AOR = 7.78, 95% CI = 1.48–40.83) and shortage of supplies (AOR = 60.8, 95% CI = 1.07–32.83) were significantly associated predictors of laboratory service interruptions.

**Conclusion:**

Neither of the hospital's laboratories met the required score of the SLIPTA standard for quality clinical laboratory services. Moreover, laboratory service interruptions were very common in the two public hospitals in which clinical chemistry, serology and hematology tests were the most frequently interrupted. Shortage of reagents and supplies and inefficient laboratory management are the major causes of service interruptions. So, policymakers and other stakeholders should support continuous quality improvement for a better patient clinical outcome.

## Introduction

### Background

Health laboratory services play a pivotal role in disease prevention, diagnosis, management and monitoring (1). Despite technological advancements in clinical laboratory services in many developed countries, laboratory services have not met the minimum standard in resource-limited nations. This is because there are a limited number of skilled medical laboratory professionals, poor infrastructures, and limited availability of advanced equipment (2). According to the World Health Organization (WHO), there is a global shortage of healthcare professionals, with an estimated 18 million healthcare workers needed to achieve universal health coverage (3). The American Bureau of Laboratory Statistics projects a nationwide need for a 13% average increase in medical laboratory technologists and technicians between 2016 and 2026, which is nearly double the current 7% underlying average increase in all occupations (4). Furthermore, the lack of national and local medical laboratory policies leads to common laboratory service interruptions in low-income countries like Ethiopia (5). Clinical laboratory risk analysis shows that multiple risks may occur due to chemicals in the laboratory environment, biological factors, differences in work equipment, and the inherent dangers of biomedical research (6). As a result, efforts to achieve equality in healthcare services remain important policy concerns, particularly in developing countries (7).

Quality in medical diagnostics is defined as the reliability, accuracy, and timeliness of laboratory test results. This needs quality management practices which focus on ensuring 12 quality essentials: personnel, organization, purchasing and inventory, equipment, process control, documents and records, information management, occurrence management, assessment, facility and safety, process improvement, and customer services (8). Poor quality laboratory services often lead to unnecessary expenditures, misery in human lives and suffering, and overuse of antibiotics for inappropriate clinical circumstances which result in the emergence of drug-resistant microorganisms (9). In addition, interruption of laboratory service may cost a human life, create a negative impact on an organization's reputation, and cause revenue loss (10). Furthermore, laboratory service interruptions are not compatible with ongoing hospital operations and would have an immediate negative impact on the high-acuity clinical services that are essential for acute care hospitals (11). In sub-Saharan Africa, the major challenge in delivering quality health services is lack of reliable medical laboratory services (12). The most common challenges are a lack of internal quality control materials and other reagents, inconsistent electric power supply, and a limited number of trained laboratory personnel. In addition, inflated equipment maintenance and calibration costs are obstacles in delivering a timely laboratory service (13).

Some studies revealed that laboratory service quality in Ethiopia remains insufficient due to several reasons, including lack of quality and adequate equipment, non-adherence to standard operating procedures, no continuing professional development, unavailability of adequate supplies and reagents, poor customer service management, lack of regular internal and external quality assessment activity, interrupted laboratory service, absence of result verification, and inadequate laboratory safety (14).

In Ethiopia, clients are often not satisfied with clinical laboratory services due to poor service delivery and interruption (15). However, the quality level of health laboratory services and frequency of service interruptions are not sufficiently evaluated in hospitals in Eastern Ethiopia. Therefore, this study aimed to assess the level of health laboratory service quality, frequency of service interruption, and its predictors in public hospitals in Harar town, eastern Ethiopia.

## Materials and methods

### Study settings

The study was conducted in two public hospitals situated in Harar town, eastern Ethiopia. Harar is the capital city of Harari regional state. The city is one of the oldest cities in Ethiopia, and is 526 km away from Addis Ababa, the capital city of Ethiopia. According to the Central Statistical Agency forecast of 2022, the population of Harari regional state was 276,431, of whom 139,576 were male and 136,855 female (16). Harari region has two public hospitals, one military hospital and one private hospital. The public hospitals, namely Hiwot Fana Specialized University Hospital (HFSUH) and Jugel General Hospital (JGH), were selected for this study as they provide multidimensional healthcare services.

### Study design and period

A facility-based cross-sectional study was conducted from January to April 2024.

### Study population

The study populations were the HFSUH and JGH laboratories in Harar town and laboratory professionals who were working in these public hospitals during the data collection period.

### Inclusion and exclusion criteria

#### Inclusion criteria

The study included HFSUH and JGH laboratory tests that were performed in the hospitals 3 months before initiation of the study. Laboratory professionals working at least in the previous 6 months in the selected hospital were also included in the study.

#### Exclusion criteria

The laboratory tests that were not performed within the previous 3 months prior to initiation of the study, laboratory professionals who worked for less than 6 months or were on leave during the study period, and involuntary laboratory professionals were excluded from the study.

### Sample size determination

We estimated the sample size for analyzing predictors of service interruptions using a single population proportion formula by taking 28% laboratory service interruption in hospitals in Addis Ababa (17). Level of significance (*α*) = 0.05, marginal error (*d*) = 5%, *Z* (*α*/2) = *Z*-score at 95% confidence interval (CI) = 1.96. Based on the formula the sample size (*n*) would be 272. However, the total number of laboratory professionals working in the selected hospitals was 59. Therefore, we employed a sample size correction factor and the final sample size became 54 after considering a 10% non-response rate.

### Data collection method

A convenient sampling technique to assess the level of health laboratory service quality was to collect primary data using the WHO Standardized Stepwise Laboratory Improvement Process Towards Accreditation (SLIPTA) checklist version 2:2015 (18). The WHO SLIPTA checklist, version 2:2015, evaluates laboratory quality system performance based on 12 essentials, and was established to strengthen clinical and public health laboratory systems in developing countries and to achieve the requirements of ISO 15189.

The study also utilized a checklist adopted from the Ethiopian Hospital Standard Transformation Guideline (EHSTG), which had originally been devised to determine the magnitude of laboratory service interruption and its predictors in the selected public hospitals (19). The second checklist adopted from EHSTG was used to assess the status of laboratory service interruption. The assessment was conducted in all laboratory units including clinical chemistry, parasitology, urinalysis and body fluid analysis, hematology, serology, mycology, and bacteriology services. The checklist was used to collect interrupted tests based on the number of tests expected to be done for three consecutive months in both hospitals. Moreover, a structured self-administered questionnaire was used to get data for the socio-demographic characteristics and professional experiences of the study participants.

The data was collected by four trained laboratory professionals and supervised by senior experienced medical laboratory professionals who are certified in Laboratory Quality Management and Strengthening Laboratory Management Toward Accreditation.

### Measurements

The level of laboratory service interruption was measured across three consecutive months using a standard checklist adopted from the EHSTG. A “tick” was recorded if the test was done or not interrupted and “x” if the test was not done or interrupted. The test counted as interrupted if the test was not done for 1 or more than 1 day. Then calculation is done as follows:

 • Percentage of interrupted tests in each hospital = (sum of interrupted tests from all services/number of tests expected to be done in the hospital) × 100.
 • Percentage of total average number of interrupted tests in public hospital = (average number of interrupted tests in all public hospitals/number of expected tests to be done in public hospitals) × 100.

The level of quality laboratory services is determined based on the total quality management practice of the hospital laboratory. The SLIPTA Checklist contains 12 main sections that have a total of 275 points.

#### Dependent variable

 • Level of laboratory service quality.
 • Magnitude of laboratory service interruption.

#### Independent variables

Socio-demographic characteristics, professional levels and experiences, and quality essentials: document and records, management review, organization and personnel, client management and customer service, equipment, evaluation, and audits, purchasing and inventory, information management, process control, identification of non-conformities, corrective and preventive action, occurrence management and process improvement, facilities, and safety. Predictors of laboratory service interruptions: resource, management support, infrastructure, natural disaster, and emergency.

### Operational definitions of variables

Laboratory service interruption: if the test was performed 3 months prior to initiation of the study in the hospital and it is not done for the one or more days during the study period, it counted as interrupted (19).

Laboratory service quality: the degree to which a set of inherent characteristics fulfills requirements (8).

Management review: top management reviews the organization’s quality management system at planned intervals to ensure its continuing suitability, adequacy, effectiveness, and alignment with the strategic direction of the organization (20).

Resources: means availability of budget, reagent and supply, quality equipment, calibration and control, and equipment maintenance with spare parts (8).

### Quality control

The checklists and questionnaire were pre-tested in Haramaya General Hospital with 5% of the total calculated sample size of laboratory professionals to identify ambiguity and potential challenges. In the actual study the raw data was checked every day for completeness and consistency. Training was given to data collectors on the purpose of the study and its method of data collection.

### Method of data analysis

After checking the completeness and consistency of the collected data, the data were entered and analyzed using SPSS software version 26. Descriptive statistics were performed to calculate, the means, percentage of interruption of tests in each laboratory unit, and the frequency of event occurrences. The SLIPTA Checklist contains 12 main sections that have a total of 275 points. Each item has been awarded a point value of 2, 3, or 5 points based on relative importance and/or complexity. Service levels were evaluated based on the final scores of 12 quality system essential elements. Those laboratories that scored <55%, 55%–64%, 65%–74%, 75%–84%, 85%–94%, and ≥95% were awarded 0, 1, 2, 3, 4, and 5 stars, respectively (18). Bivariate and multivariable logistic regressions were used to identify the association between outcome and independent variables. Independent variables with *p*-value <0.25 were carried on into a multivariable logistic regression model. Independent variables that had *p*-values <0.05 with 95% CI were considered to be a significant association. The major findings were summarized and presented in tables and figures.

### Ethical consideration

The principle of health research ethics was maintained, and ethical clearance was obtained from Haramaya University's College of Health and Medical Science and the Institutional Health Research Ethical Review Committee. The permission letter and informed, voluntary, and signed consent were taken from the HFSUH and JGH directors. Similarly, informed written signed consent was obtained from each study participant after explaining the research purpose, procedure, period, possible risk, and benefit. Every study participant had the right to make a decision about participation in the study and participants who were not willing to participate in the study were not forced to participate. All responses were coded to maintain confidentiality of the respondents for the information given.

### Information dissemination

The result of this study was submitted to Haramaya University, College of Health and Medical Sciences, and the School of Medical Laboratory Sciences as well as Harari Regional State Health Office, HFSUH, and JGH to inform the policies and programs.

## Results

### Level of laboratory service quality

According to EHSTG, 76 types of tests are expected to be performed in a specialized hospital whereas 68 types of tests are anticipated in a general hospital (19). In our study, 72 (94.7%) and 64 (94%) types of tests are available in HFSUH and JGH, respectively. Both hospital laboratories have clinical chemistry, parasitology, urinalysis and body fluid analysis, hematology, serology, mycology, and bacteriology service units. According to the SLIPTA Checklist, which contains 12 main sections, our assessment findings indicate that from the two laboratories, none of them met the required standard. The mean score of the two hospitals was 168.5 (61%). The mean laboratory service quality scores of HFSUH and JGH laboratories were 212 (77%) and 127 (46.1%), respectively.

**Figure 1:**
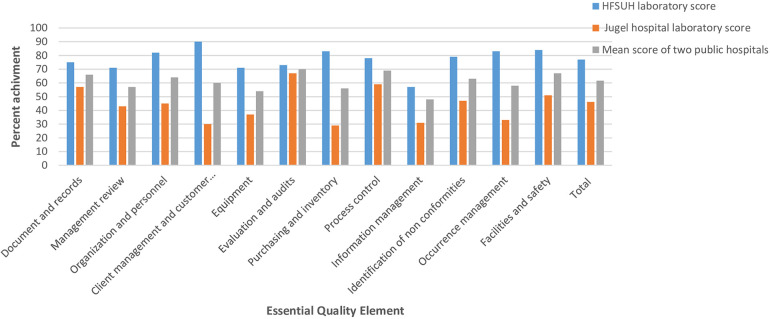
The percent score recorded based 12 essential quality elements in public hospitals laboratories, Harar town, January 2024 to 8 April 2024.

The least mean service quality scores were observed in information management (47.60%), equipment (51.4%), purchasing and inventory (56.25%), management review (57.12%), and occurrence management (58.30%) in the two hospitals. Mean scores above average were recorded for process improvement (69%), evaluation, and audits (70%). Considerably greater scores for the 12 essential quality elements were observed in the HFSUH laboratory than the JGH laboratory, but none of them met the SLIPTA recommended maximum mean score (Figure 1).

### Magnitude of laboratory service interruption

The total numbers of interrupted tests in public hospitals were assessed based on existing laboratory services. The existing laboratory service performance was followed on a daily basis for 90 days to detect potential laboratory test interruptions. The data collectors recorded the type of test interrupted, the time of interruptions and the reason for the interruptions. Of all 72 tests in the two hospitals, on average 31 (43.05%) tests were interrupted at least for 1 day. Clinical chemistry test interruption accounted for 16 (51.6%) followed by 7 in serology (22.6%) and 4 in hematology (12.9%). Parasitological and urine analysis tests were the least interrupted, with parasitology test interruption occurring only for 1 day and no interruption of urinalysis.

The laboratories at both hospitals did not provide continuous diagnostic services for all available tests according to EHSTG. In the HFSUH laboratory 19/76 (25%) laboratory tests were interrupted at least for 1 day; accounting for the highest interruptions were of serology tests (6/9, 31.6%), hematology tests (5/19, 26.3%) and clinical chemistry (5/19, 26.3%). Of the 68 tests, 43 (63.2%) at JGH laboratory services were interrupted for at least 1 day, in which clinical chemistry tests (28/43, 65.1%), serology (7/43, 16.3%), and hematology (3/43, 7%) accounted to the highest proportions of interruptions.

We also assessed the common reasons for laboratory service interruptions. Cardiac function tests, thyroid stimulating hormone (TSH) and hematological tests were interrupted due to a shortage of reagents. Serological tests, occult blood, and potassium hydroxide (KOH) tests were discontinued mainly due to a shortage of test kits in the HFSUH laboratory. In the JGH laboratory all clinical chemistry tests and some hematological tests were interrupted due to a shortage of reagents. Besides occult blood, KOH tests, and serological tests were interrupted due to a lack of test kits. In this hospital cerebrospinal fluid (CSF) analysis, India ink, culture, and sensitivity were not started in the hospital. Chemistry tests for blood glucose, lipid profiles, liver function tests, renal function tests, and cardiac function tests had a longer interruption, starting after 63 days of interruption, but albumin was interrupted again, due to a failure to pass calibration.

Out of 31 laboratory service interruptions in these public hospitals, 27 (87%) were due to shortages of reagents and test kits. KOH tests, India ink, and hematology tests (bleeding time, reticulocyte count, and prothrombin time) were interrupted for 90 days in both hospitals. HFSUH laboratory experienced interruptions in cardiac function tests (lactate dehydrogenase, creatinine kinase subunit M and B, and creatinine phosphokinase) for 90 days and from hormonal tests TSH was interrupted for 90 days. Jugel General Hospital laboratory experienced interruptions in electrolyte tests, cardiac function tests, and hormonal tests for 90 days each. HFSUH laboratory experienced no interruptions in CSF analysis and electrolyte tests. JGH laboratory experienced a 90-day interruption in CSF analysis and electrolyte tests, and also 63-day interruptions in liver function tests and renal function tests (Table 1).

**Table 1:** Number of days laboratory services interruptions and reasons of interruptions in public hospitals in Harar, January 2024 to 8 April 2024.

Types of laboratory services		HFSUH laboratory		JGH laboratory	
		No of days of interruption	Reasons of interruption	No of days of interruption	Reasons of interruption
Clinical chemistry	Blood glucose	0	—	63	Lack of reagents
	Lipid profiles (cholesterol, triglyceride, LDL, HDL)	0	—	63	Lack of reagents
	Electrolytes (Na+, K+, Cl−)	0	—	90	Lack of reagents
	Liver function tests (ALKP, AST, ALT, GGT, total bilirubin, direct bilirubin, total protein)	0	—	63	Lack of reagents
	Albumin	0	—	90	Lack of reagents, calibration failure
	Renal function tests (urea, creatinine, uric acid)	0	—	63	Lack of reagents
	Cardiac function test (LDH, CK-MB, CPK)	90	Lack of reagents	63	Lack of reagents
	Cardiac function test (troponin)	40	Lack of reagents	63	Lack of reagents
	Hormonal test (TSH)	60	Lack of reagents	63	Lack of reagents
	Hormonal test (T3, T4, FSH, LH)	0	—	90	Lack of reagents
	Hormonal test (testosterone, progesterone, prolactin)	0	—	Not applicable	—
Parasitology test	Stool microscopy, blood film/other hemoparasites	0	—	0	—
	Occult blood	14	Lack of test kits	90	Lack of test kits
Urine and body fluid analysis	Urinalysis	0	—	0	—
	CSF analysis	0	—	90	Not started in the hospital
Mycology	KOH test	90	Lack of test kits	90	Lack of test kits
	Fungal culture	0	—	Not applicable	—
Hematology	Total WBC count, differential white cell count, peripheral blood film, ESR, hematocrit, platelet count	0	—	0	—
	Bleeding time, reticulocyte count, prothrombin time	90	Replaced by another tests	90	Lack of reagents
	Hb electrophoresis, lupus erythematosus	90	Not done in the hospital	Not applicable	—
Serology	*Helicobacter pylori*	0	—	10	Lack of test kits
	HCV	0	—	10	Lack of test kits
	HBsAg	0	—	15	Lack of test kits
	Toxoplasma latex, TPHA, troponin	90	Lack of test kits	90	Lack of test kits
	RPR, Widal and Weil–Felix, blood group, cross match and compatibility test, HCG, RF, CRP	0	—	0	—
	ASO	0	—	25	Lack of test kits
	HIV test	0	—	22	Lack of test kits
	CD4 count, viral load	7	Lack of test kits	Not applicable	—
Bacteriology	Gram stain	0	—	0	—
	Ziehl–Neelsen stain	0	—	0	—
	India ink	90	Lack of test kits	90	Lack of test kits
	Culture and sensitivity	0	—	90	Not started in the hospital

ALKP, alkaline phosphatase; AST, aspartate aminotransferase; CK-MB, creatinine kinase in blood; CPK, creatinine phosphokinase; ESR, erythrocyte sedimentation rate; FSH, follicle-stimulating hormone; GGT, gamma-glutamyl transferase; HCG, human chorionic gonadotropin; HDL, high-density lipoprotein; LDL, low-density lipoprotein; LDH, lactate dehydrogenase; LH, luteinizing hormone; RPR, rapid plasma regain; TPHA, treponema pallidum hemagglutination assay.

### Predictors of laboratory service interruption

In total, 52 laboratory professionals participated, with a response rate of 96.3% in which 28 (53.8%) were male and 24 (46.2%) female. The highest number (22, 42.3%) of participants fell between the 30 and 35 years age group with a mean age of 36.19 years (SD = 6.38, a range of 24–54 years). The participants’ educational backgrounds are diverse; 40 (76.9%) of them have BSc degrees, 8 (15.4%) have a diploma or certificate, and 4 (7.7%) have a master's degree. In addition, 23 (44.2%) had 6–10 years’ work experience as laboratory professionals and the majority of the respondents 37 (71.1%) were working in a current hospital for more than 6 years. The majority of participants (47, 90.4%) identified as ordinary laboratory staff and a few participants (5, 9.6%) were occupying specialized positions such as safety officers, quality officers, and laboratory heads (Table 2).

**Table 2:** Socio-demographic characteristics of study participants in public hospitals, Harar, 8 January 2024 to 8 April 2024.

Variables		Frequencies	Percent
Sex of laboratory professionals	Male	28	53.8
	Female	24	46.2
Age of laboratory professionals	24–29	5	9.6
	30–35	22	42.3
	36–41	18	34.6
	42–47	1	
	48–55	6	11.5
Level of highest education	Diploma\certificate	8	15.4
	First degree	40	76.9
	Masters	4	7.7
Total service year as a lab professional	1–5	3	5.8
	6–10	23	44.2
	11–15	19	36.5
	16–20	2	3.8
	21–25	2	3.8
	26–30	3	5.8
Total service year in current hospital	1–5	15	28.8
	6–10	20	38.5
	11–15	11	21.2
	16–20	1	
	21–25	5	9.6
Position	Staff as lab professionals	47	90.4
	Safety officer	1	
	Quality officer	2	3.8
	Laboratory head	2	3.8

According to this study, 37 (71.1%) of the participants responded that their laboratory services were continuously available while the rest (12, 23.1%) of them indicated that their laboratory services were interrupted frequently. Nearly 81% (42) of them were dissatisfied with their salary and 37 (71.2%) of them blamed failings on a high workload (Table 3). In addition, 39 (75%) of them responded that there was lack of adequate training in their hospital laboratory while 37 (71.2%) stated that their laboratory had inadequate working spaces. Concerning continuous power supply, 36 (69.2%) participants reported that their laboratory obtained continuous power supply and 35 (67.3%) water supply, whereas 36 (69.2%) laboratory professionals responded that backup power was unavailable when the electric power went off.

**Table 3:** Response characteristics of laboratory professionals on laboratory service interruption in public hospitals in Harar, 8 January 2024 to 8 April 2024.

SN	Predictors	Study participants response	
		Yes	No
Individual factors			
1	Satisfaction with current salary	10 (19.2%)	42 (80.8%)
2	Availability of safety equipment and use in the laboratory	19 (36.5%)	33 (63.5%)
3	Availability of any immunization	23 (44.2%)	29 (55.8%)
4	Delivery of adequate training	13 (25%)	39 (75%)
5	Workload	37 (71.2%)	15 (28.8)
Infrastructure			
6	Availability of enough space	15 (28.8%)	37 (71.2%)
7	Availability of sufficient human resources for laboratory work	14 (27%)	38 (73%)
8	Availability of continuous, functional electric power	36 (69.2%)	16 (30.8%)
9	Availability of continuous, functional water	35 (67.3%)	17 (32.7%)
10	Availability of backup power if electric power interrupted	16 (30.8%)	36 (69.2%)
Resource			
11	Availability of sufficient budget	14 (27%)	38 (73%)
12	Availability of sufficient reagents and supplies on time	24 (46.2%)	28 (53.8%)
13	Adequacy of laboratory equipment	11 (21.2%)	41 (78.8%)
14	Availability of trained lab professionals that can run laboratory equipment	37 (71.2%)	15 (28.8%)
15	Availability of malfunctioning equipment	30 (57.7%)	22 (42.3%)
16	Availability of equipment maintenance on time	27 (53%)	25 (48%)
17	Availability of additional laboratory equipment used as backup	24 (46.2%)	28 (53.8%)
Top management support			
18	Management response for questions related to laboratory service	16 (30.8%)	36 (69.2%)
19	Allocation of laboratory budget at the beginning of the year	20 (38.5%)	32 (61.5%)
20	Availability of laboratory annual plan	38 (73.1%)	14 (26.9%)
21	Following essential supplies, reagents, and equipment on time	35 (67.3%)	17 (32.7%)
22	Presence of continuous communications with upper management about laboratory service	31 (59.6%)	21 (40.4%)
Natural disaster and emergency			
23	Occurrence of natural disaster or emergency (pandemic, fire accident, flood, laboratory floor subsidence) in the hospital	12 (23.1%)	40 (76.9%)
24	Interruption of lab services	15 (28.8%)	37 (71.25%)

Three-fourth (38%) of the study participants believed that their clinical laboratory department had the insufficient budget. In addition, 38 (73%) of them indicated a shortage of human resources. Forty-one (78.8%) laboratory professionals understood that laboratory equipment was inadequate and 30 (57.7%) of them described malfunctioning equipment while 25 (48%) participants thought that equipment did not receive maintenance on time. According to this study concerning top management support, 36 (69.2%) laboratory professionals indicated that there were delayed responses from upper management for questions related to laboratory service, and 32 (61.5%) suggested there were no smooth communication systems.

### Multivariable logistic regression analysis

In the bivariate analysis variables like insufficient human resources, unavailability of sufficient reagents and timely delivery of supplies, inadequate laboratory equipment, unavailability of equipment maintenance on time, unavailability of additional laboratory equipment to be used as a backup, occurrence of natural disaster or emergency, and delayed response of upper management response for questions related to laboratory service were all found to be significant. Variables with a *p*-value <0.25 were selected as candidates for multivariable logistic regression analysis. In multivariable logistic regression analysis variables like unavailability of sufficient reagents (*p* = 0.04) and supplies on time and lack of upper management response to laboratory service reports (*p* = 0.015) were statistically associated with laboratory service interruption (Table 4).

**Table 4:** Bivariate and multivariable logistic regression analysis of predictors of laboratory services interruptions in public hospitals laboratories in Harar, January 2024 to 8 April 2024.

Variables		Laboratory services interruptions		Logistic analysis			
		Yes	No	COR (95% CI)	*p*-value	AOR (95% CI)	*p*-value
Availability of safety equipment (lab coat, glove, eye wash, etc.) and use in the laboratory	Yes	6	13	1	0.74		
	No	9	24	1.23 (0.36–4.23)			
Availability any vaccine	Yes	6	17	1	0.70		
	No	9	20	0.78 (0.23–2.65)			
Delivery of adequate training	Yes	5	8	1	0.38		
	No	10	29	0.55 (0.15–2.08)			
Workload	High	9	28	1	0.26		
	Medium	6	9	2.074 (0.58–7.44)			
Availability of enough space	Yes	5	10	1	0.65		
	No	10	27	0.74 (0.20–2.71)			
Availability of continuous, functional electric power	Yes	9	27	1	0.361		
	No	6	10	1.8 (0.51–6.36)			
Availability of continuous, functional water	Yes	9	26	1	0.48		
	No	6	11	1.58 (0.45–5.50)			
Availability of backup power if electric power interrupted	Yes	5	11	1	0.80		
	No	10	26	0.85 (0.23–3.06)			
Availability of sufficient budget	Yes	6	8	1	0.51		
	No	9	29	0.64 (0.17–2.38)			
Availability of sufficient human resources for laboratory work	Yes	6	8	1	0.182	1	0.99
	No	9	29	0.41 (0.11–1.51)		0.99 (0.15–6.38)	
Availability of sufficient reagents and supplies on time	Yes	10	14	1	0.07	1	0.04^a^
	No	5	23	0.30 (0.089–1.078)		6.8 (1.07–32.823)	
Adequacy of laboratory equipment	Yes	5	6	1	0.17	1	0.37
	No	10	31	0.39 (0.10–1.55)		2.38 (0.35–16.18)	
Availability of trained lab professionals that can run laboratory equipment	Yes	10	27	1	0.65		
	No	5	10	1.35 (0.37–4.93)			
Availability of malfunctioning equipment	Yes	10	20	0.41 (0.49–5.95)	0.41		
	No	5	17	1			
Availability of equipment maintenance on time	Yes	10	17	1	0.18	1	0.516
	No	5	20	0.43 (0.12–1.88)		1.68 (0.35–7.97)	
Availability of additional laboratory equipment used as backup	Yes	5	19	1	0.24	1	0.414
	No	10	18	2.11 (0.61–7.39)		0.48 (0.08–2.83)	
Upper management response for questions related to laboratory service	Yes	9	7	1	0.01	1	0.015^a^
	No	6	30	0.16 (0.05–0.58)		7.78 (1.48–40.83)	
Allocation of laboratory budget at the beginning of the year	Yes	5	15	1	0.63		
	No	10	22	1.36 (0.389–4.80)			
Availability of laboratory annual plan	Yes	8	16	1	0.51		
	No	7	21	0.67 (0.2–2.22)			
Following essential supplies, reagents, and equipment on time	Yes	10	23	1	0.76		
	No	5	14	0.82 (0.23–2.90)			
Presence of continuous communications with upper management about laboratory service	Yes	9	22	1	0.97		
	No	6	15	0.98 (0.29–3.33)			
Occurrence of natural disaster or emergency	Yes	6	6	3.44 (0.89–13.33)	0.07	1	0.20
	No	9	31	1		0.27 (0.04–2.04)	

COR: crude odds ratio.

^a^ Statistical significant association.

## Discussion

Based on the WHO Regional Office for Africa SLIPTA checklist, the mean score of the two public hospital laboratories in Harar was 168.5 (61%). However, HFSUH and JGH laboratories had quality essential scores of 212 (77%) and 127 (46.1%), respectively. The mean score of the two public hospital laboratories in Harar was higher than the study conducted in Addis Ababa, which showed that the average score for government laboratories was 78.2 (31.2%) points (21). The HFSUH laboratory score was higher than the study conducted in Addis Ababa, which showed that the overall implementation of the 12 quality system essentials was <35% (22), but the JGH laboratory score was similar to study conducted in Addis Ababa, which indicated that 9 of them had less than 55% (23). This might be due to a lack of training for laboratory professionals to implement a laboratory quality management system (24).

According to the current study, the HFSUH laboratory scored more than 50% of the values for all essential quality elements. In client management (90%), purchasing and inventory (83.3%), occurrence management (83.3%), and facility and safety (83.3%) scored the highest value of quality elements. This finding is higher than the study conducted in Addis Ababa, which scored values slightly over 50% only for equipment, purchasing, inventory, and information management (22). JGH laboratory scored more than 50% only in three essential quality elements like document and record (57.1%), process control (59.4%), and evaluation and audits (66.6%). Purchasing and inventory (29.2%), client management (30%), occurrence management (33.3%), equipment (37%), information management (38.1%), management review (42.9%), and organization and personnel (45.5%) were the least scored quality elements, which is similar with the study conducted in Addis Ababa (23) where occurrence management, corrective action, management review, information management, and process improvement were the least scored quality elements, scoring 23%, 30%, 40%, 49%, and 53%, respectively. This might be due to a lack of continuous communication with upper management about laboratory services (25).

According to the current study, there were service interruptions in both hospitals' laboratories. This may have various significant consequences, such as delays in diagnosing medical conditions, which can result in delayed treatments and potentially worsen patient outcomes, which may result in increased complaints from clinicians and customers (13). This may also increase healthcare costs; patients and healthcare providers may lose trust in the healthcare system, leading to potentially incorrect diagnoses or treatments (26).

According to our study, on average 31 (43.05%) of the tests in the two studied hospitals were interrupted. Clinical chemistry tests were highly interrupted which accounts for 16 (51.6%) of total interruption (31), followed by serology (7, 22.6%) and hematology (4, 12.9%). This is higher than in studies conducted in Gaza Strip, Palestine, and in the Democratic Republic of Congo, where the most common tests—clinical chemistry, hematology, and bacteriology, followed by parasitology were continuously available in all laboratories—though serology was infrequent in all laboratories (27). The other study conducted in Addis Ababa (17) indicates that in typical public hospitals, usually 17 (23%) of tests were interrupted for 76 (84%), days which is lower than our study. In the current study, clinical chemistry, hematology, and serology were frequently interrupted which is comparable with the study conducted in Addis Ababa by Mulu. This might be due to insufficient reagents and supplies, delayed response of upper management for questions related with laboratory services, inappropriate equipment maintenance, and shortage of budget allocation (28).

On the other hand, our study finding is consistent with the study conducted in the Guragae area of southern Ethiopia in which serious gaps in the availability of necessary testing were found in all facilities examined. The essential diagnostic tests including C-reactive protein, lipid profile, amylase and lipase, troponin/I, hepatitis B virus antigen, specific IgM antibodies to hepatitis B virus core antigen, glucose dehydrogenase-6-phosphate activity, and rapid HIV/p24 tests were interrupted. However, basic diagnostic services such as urine tests, random blood glucose tests, smear microscopy, and some serological tests were provided in all primary care units (29). Another study conducted in Addis Ababa indicated that all laboratory facilities had at least one or more basic fine needle aspiration cytology (FANC) laboratory test interruption for more than a day within the last 1 year (30). This indicates that these health facilities had a limited number of essential laboratory equipment, reagents, and supplies to perform all expected laboratory tests (31).

According to the current study, unavailability of sufficient reagents and supplies was the major predictor that was significantly associated with the interruptions of laboratory services. A laboratory with insufficient reagents and supplies available is about 6.8 times more likely to interrupt laboratory services than a hospital with sufficient reagents and supplies available on time (AOR = 6.8, *p* = 0.039, 95% CI = 1.07–32.83). Hospitals in which upper management do not respond immediately to laboratory service interruption report were found 7.7 times more likely to interrupt laboratory services than hospitals in which upper management respond immediately (AOR = 7.78, *p* = 0.015, 95% CI = 1.481–40.83). This study finding is consistent with studies conducted in Sri Lanka (32) and Malawi (33) which indicated that laboratory services were interrupted by shortages of reagents. Another study conducted in Addis Ababa indicated that laboratory tests were interrupted due to a shortage of reagents (30). This might be due to a lack of adequate budget allocation (31). In our study, upper management that did not respond immediately to questions related to laboratory service was another factor significantly associated with the interruptions of laboratory services. Similarly, the study in Addis Ababa (24) found that lack of top management response was a major reason for service interruption. This might be due to a lack of continuous communications with upper management about laboratory service to deliver continuous laboratory service (34).

### Limitations of the study

For the financial reason, the study was conducted only in two public hospitals, and the laboratory service interruptions for less than 1 day were not also considered.

## Conclusion and recommendation

The findings from the current study revealed that laboratory service quality in public hospitals did not meet the required maximum international standard. There are also frequent laboratory service interruptions, particularly in clinical chemistry, serology, and hematology tests. Upper management that did not respond immediately to questions related to laboratory service, unavailability of sufficient reagents, and supplies are the major predictors of these interruptions. Therefore, policymakers should focus on developing strategies to improve the quality of laboratory services. This could include delivering adequate training to laboratory professionals to implement, evaluate and identify areas for improvement, and track progress over time to improve the quality of laboratory services, since the laboratory with the lowest star rating experienced a high percentage of interruptions. Laboratory services should be consistently available. So, upper management of hospitals and regional health bureaus should give immediate responses to questions related to laboratory services and solve the problem related with the availability of sufficient reagents and supplies to improve laboratory service interruptions. Additional detailed studies should be conducted at the national and regional levels, including health centers and private hospital laboratories.

## Data Availability

The raw data supporting the conclusions of this article will be made available by the authors, without undue reservation.
